# Health Risk Associated with Exposure to PM_10_ and Benzene in Three Italian Towns

**DOI:** 10.3390/ijerph15081672

**Published:** 2018-08-06

**Authors:** Antonella De Donno, Mattia De Giorgi, Francesco Bagordo, Tiziana Grassi, Adele Idolo, Francesca Serio, Elisabetta Ceretti, Donatella Feretti, Milena Villarini, Massimo Moretti, Annalaura Carducci, Marco Verani, Silvia Bonetta, Cristina Pignata, Silvia Bonizzoni, Alberto Bonetti, Umberto Gelatti, on behalf of the MAPEC_LIFE Study Group

**Affiliations:** 1 Department of Biological and Environmental Science and Technology, University of Salento, via Monteroni 165, 73100 Lecce, Italy; antonella.dedonno@unisalento.it (A.D.D.); mattia.degiorgi@unisalento.it (M.D.G.); tiziana.grassi@unisalento.it (T.G.); adele.idolo@unisalento.it (A.I.); francesca.serio@unisalento.it (F.S.); 2 Department of Medical and Surgical Specialties, Radiological Sciences and Public Health, University of Brescia, Viale Europa 11, 25123 Brescia, Italy; elisabetta.ceretti1@unibs.it (E.C.); donatella.feretti@unibs.it (D.F.); umberto.gelatti@unibs.it (U.G.); 3 Department of Pharmaceutical Sciences, University of Perugia, Via del Giochetto, 06122 Perugia, Italy; milena.villarini@unipg.it (M.V.); massimo.moretti@unipg.it (M.M.); 4 Department of Biology, University of Pisa, Via S. Zeno 35/39, 56126 Pisa, Italy; annalaura.carducci@unipi.it (A.C.); marco.verani@unipi.it (M.V.); 5 Department of Public Health and Pediatrics, University of Torino, Piazza Polonia 94, 10126 Torino, Italy; silvia.bonetta@unito.it (S.B.); cristina.pignata@unito.it (C.P.); 6 Comune di Brescia, Piazza Repubblica 1, 25100 Brescia, Italy; sbonizzoni@comune.brescia.it; 7 Centro Servizi Multisettoriale e Tecnologico–CSMT Gestione Scarl., via Branze 45, 25123 Brescia, Italy; a.bonetti@csmt.it

**Keywords:** health risk assessment, increased cancer risk, particulate matter, benzene, exposure, air pollution, MAPEC_LIFE study

## Abstract

Air pollution in urban areas is a major concern as it negatively affects the health of a large number of people. The purpose of this study was to assess the inhalation health risk for exposure to PM_10_ and benzene of the populations living in three Italian cities. Data regarding PM_10_ and benzene daily measured by “traffic” stations and “background” stations in Torino, Perugia, and Lecce during 2014 and 2015 were compared to the limits indicated in the Directive 2008/50/EC. In addition, an inhalation risk analysis for exposure to benzene was performed for adults and children by applying the standard United States Environmental Protection Agency’s (USEPA) methodology. The levels of PM_10_ detected in Torino exceeded the legal limits in both years with an increased mean concentration >10 µg/m^3^ comparing with background station. Benzene concentrations never exceeded the legislative target value. The increased cancer risk (ICR) for children exposed to benzene was greater than 1 × 10^−6^ only in the city of Torino, while for adults, the ICR was higher than 1 × 10^−6^ in all the cities. The results suggest the need for emission reduction policies to preserve human health from continuous and long exposure to air pollutants. A revision of legal limits would also be recommended.

## 1. Introduction

Outdoor air pollution is one of the most important environmental problem in both industrialized and developing countries, and in 2013, it was classified by the International Agency for Research on Cancer (IARC) as carcinogenic to humans (Group 1) [1]. It impacts over 600 million people living in urban areas who are exposed to dangerous levels of pollutants generated mainly by vehicular traffic, industrial activity, and, during the winter, by domestic heating systems.

In particular, traffic-generated urban air pollution is common among all cities and in all seasons and is rich in some of the most dangerous airborne pollutants like particulate matter (PM) and benzene. 

PM, particularly components less than 10 μm (PM_10_), is a major constituent of outdoor air pollution. It is a highly variable and complex mixture of particles and gases emitted directly from sources or formed in the atmosphere from gaseous emissions. Resuspension of soil tracked onto roads and streets, suspension from disturbed soils, resuspension of industrial dusts, construction, and coal and oil combustion are considered the main sources of PM_10_ emission in urban areas [2]. Exposure to PM has been identified as the cause of numerous health effects including cardiovascular and respiratory disease [3]. In addition, PM was recently classified as carcinogenic to humans (Group 1) because an increasing risk of lung cancer was associated with increasing levels of exposure to PM [1].

Benzene is considered one of the pollutants of most concern in urban areas [4,5,6]. It is released into the atmosphere mainly from gasoline vapors and automobile exhaust [7,8] and from 1987 is part of the group 1 of carcinogenic classification by IARC [9] because, for some decades, there has been sufficient evidence that inhalation exposure to this contaminant is associated with acute myeloid leukemia, myelodysplastic syndromes, and probably lymphoma and childhood leukemia [10].

Nevertheless, many studies have demonstrated that exposure to benzene can cause many other diseases, both acute and chronic, which can impact several human tissues or organs. The effects can also affect the central nervous system [11], the reproductive and developmental system [12,13,14], the immune system [10,15], and the respiratory system [16]. Metabolites of benzene are also involved in its toxicity [17]. Both toxic and carcinogenic effects of benzene are due to several factors such as the duration and levels of exposure, the way of exposure, and individual susceptibility factors (age, gender, lifestyle, and pre-existing disease). Therefore, due to the important toxic effect of PM and benzene, the risk level of exposed populations should be assessed early before irreversible health effects can occur with high social and health damage. 

One of the most used methods to assess the healthiness of the environmental matrices is the standard risk analysis developed in 1989 by the United States Environmental Protection Agency (USEPA) [18] and subsequently updated and integrated. The standard USEPA methodology is based on a toxicological approach and was designed to evaluate the effects on human health resulting from prolonged exposure via ingestion, inhalation, or dermal absorption to toxic substances in environmental matrices of contaminated sites. It is the tool indicated by several guidelines for management of polluted sites, such as Italian legislation (Legislative Decree No. 152/2006) [19], to establish whether a potentially contaminated site requires remediation to reduce the health risk to acceptable levels. However, it was also applied in many studies conducted on a larger scale to assess the health risk associated with widespread environmental pollution, such as airborne pollution in urban areas produced by vehicular traffic [20,21,22].

The aim of this research was to assess the level of exposure to PM_10_ and benzene of people living in urban areas affected by vehicular traffic of three Italian cities (Torino, Perugia, and Lecce) and their inhalation health risk.

## 2. Materials and Methods 

### 2.1. Study Design

This study was included in the “Monitoring Air Pollution Effects on Children for supporting public health policy” (MAPEC_LIFE) project (LIFE12 ENV/IT/000614) [23], a multicenter cohort study funded by the European Union’s LIFE+ Program. It aimed to evaluate, both by toxicological and epidemiologic-molecular approaches, the association between air pollution exposure and early biological effect in 6–8-year-old children living in Italian areas with varying levels of air pollution [24,25].

**Figure 1:**
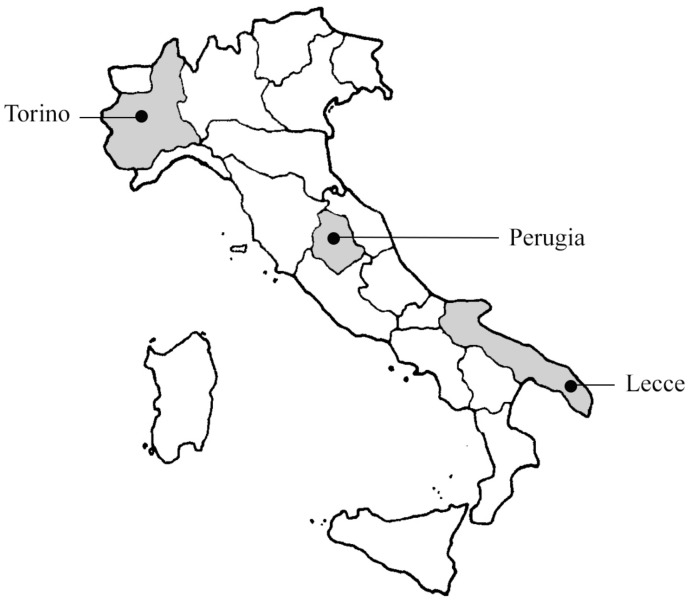
Location of the three cities involved in the study.

Within the toxicological risk assessment, an inhalation risk analysis was carried out for people living in Torino, Perugia, and Lecce on the basis of atmospheric concentrations of PM_10_ and benzene in the years 2014 and 2015. The three towns (Figure 1) are located in different areas of Italy with different environmental and socio-economic characteristics [26,27]. Torino is located in the Po valley (Northern Italy), a heavily industrialized area with the highest levels of air pollution in Europe. Perugia is located in a medium-low polluted area in central Italy. Lecce is a city in southern Italy with a low industrial development.

### 2.2. Environmental Data

Data on the concentration of PM_10_ and benzene in Torino, Perugia, and Lecce in the years 2014 and 2015 were acquired from seven permanent monitoring stations located in the urban areas of the three cities affected by vehicular traffic. Moreover, PM_10_ data were acquired from a background station located outside the urban area of each city, in a rural area far from evident sources of pollution. All the stations belonged to the air quality monitoring network of the respective Regional Agencies for the Environmental Protection (ARPA Piemonte, ARPA Umbria, and ARPA Puglia) (Table 1).

PM_10_ and benzene in each monitoring station were measured continuously respectively by gravimetric method and gas-chromatography as indicated in the Directive 2008/50/EC on ambient air quality and cleaner air for Europe. The validated hourly values of PM_10_ and benzene in each monitoring station were entered in a Microsoft Excel database and processed in order to obtain the daily averages, which in turn were used to calculate the annual average, standard deviation (SD), and 95% upper confidence limit (UCL) of the mean concentration. The values of both PM_10_ and benzene were compared to the reference values indicated in Directive 2008/50/EC [28] and in the World Health Organization’s (WHO) Air Quality Guidelines [29] in order to verify if the safe levels were exceeded. The paired sample t-test was used to detect any significantly difference (*p* < 0.01) between traffic stations and relative background station. In addition, a linear regression model between benzene and PM_10_ concentrations was performed, and the correlation coefficient was calculated in each station for whole study time by the MedCalc software, version 14.8.1 (MedCalc Software bvba, Ostend, Belgium).

### 2.3. Risk Analysis

An inhalation risk analysis based on toxicological approach was performed for exposure to benzene through the use of the procedure described in the USEPA guidelines [30] and reported in the Italian guidelines for the assessment of inhalation exposure in contaminated sites [31]. Risk analysis was conducted by assessing the non-carcinogenic and carcinogenic risk both for adults and children residing in the areas identified by the monitoring stations. Non-carcinogenic risk was evaluated with the Hazardous Quotient (HQ) defined as “The ratio of a single substance exposure level over a specified time period to a reference dose for that substance derived from a similar exposure period” [30]. It expresses whether the exposure is higher (>1) or lower (<1) than the maximum exposure in which there is no effect on human health and was calculated according to the following equation:

(1)  HQ =EC/RfC 

where RfC (mg/m^3^), the inhalation reference concentration, is the estimate of a continuous inhalation exposure for the human population (including sensitive subgroups) that is likely to be without appreciable risk of deleterious effects during a lifetime. EC (μg/m^3^) is the chronic exposure concentration, averaged over the exposure time for non-carcinogenic risk or over a lifetime for carcinogenic risk, calculated according to the following equation:

(2)  EC =(CA × ET × EF × ED)/AT 

where CA (μg/m^3^) is the air pollutant concentration in the exposure point corresponding to UCL of values measured in each monitoring station and in each time lapse, ET (h/day) is the exposure time, EF (days/year) is the exposure frequency, ED (years) is the exposure duration, and AT (years × 365 days/year × 24 h/day) is the averaging time of exposure.

The increased cancer risk (ICR), (the incremental probability of developing cancer over a lifetime as a result of exposure to one or more chemicals) for a receptor exposed via the inhalation pathway to benzene was estimated with the following equation:

(3)  ICR = IUR × EC 

where IUR ((μg/m^3^)^−1^) is the Inhalation Unit Risk defined as the “upper-bound excess lifetime cancer risk estimated to result from continuous exposure to an agent at a concentration of 1 μg/m^3^ in air” [30].

The toxicity values were taken from the Integrated Risk Information System’s (IRIS) toxicological database [32], which for benzene poses RfC equal to 3.00 × 10^−2^ mg/m^3^ and IUR equal to 7.80 × 10^−6^ (μg/m^3^)^−1^. 

The values of exposure factors were taken from Exposure Factor Handbook [33] by taking into account the “residential” exposure and placing ET equal to 24 h/day, EF equal to 350 days/year, ED equal to 24 years for adults and 6 years for children, AT equal to ED for non-carcinogenic risk (HQ), and 70 years for carcinogenic risk (ICR). 

## 3. Results

The annual average of PM_10_ ± SD measured in 2014 and 2015 at each monitoring station and the number of times in which the daily limit of 50 µg/m^3^ indicated by European regulation was exceeded are shown in Table 2. Rebaudengo station (Torino) registered the highest annual average and the highest number of exceedances of the daily limit value in both 2014 and 2015, while Garigliano station (Lecce) registered the lowest values. The comparison between the background station and the traffic stations in Torino showed an increased concentration of PM_10_ in urban areas of 14.52 µg/m^3^ to 18.78 µg/m^3^. These differences appeared to be significant in both years. In Perugia the differences between background and traffic stations ranged from −1.44 µg/m^3^ to 4.79 µg/m^3^, but only Cortonese Park station recorded significant increases in both years, while Fontivegge station showed a significant increase only in 2014. Finally, in Lecce, the increase of PM_10_ was significant in 2014 (1.55 µg/m^3^ to 3.00 µg/m^3^) for both traffic stations, while in 2015, the level of PM_10_ did not show appreciable differences between urban and non-urban areas.

Benzene concentration registered in all cities in 2014 and 2015 is reported in Table 3. Rebaudengo station (Torino) showed the highest annual average in both years, while Parco Cortonese station (Perugia) registered the lowest values.

**Figure 2:**
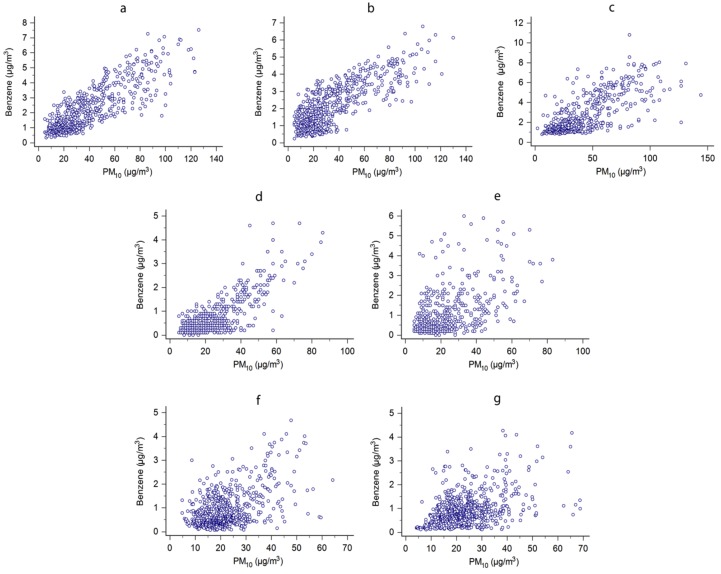
Linear regression between benzene and PM_10_ concentrations in the stations of (**a**) Consolata (*r* = 0.8473); (**b**) Rubino (*r* = 0.7986); (**c**) Rebaudengo (*r* = 0.7486); (**d**) Cortonese (*r* = 0.7839); (**e**) Fontivegge (*r* = 0.5260); (**f**) Garigliano (*r* = 0.5001); (**g**) Libertini (*r* = 0.4179).

The linear regression between benzene and PM_10_ concentrations showed a stronger correlation in Consolata station (Torino), followed by Rubino (Torino), Cortonese (Perugia), and Rebaudengo (Torino) stations, as shown in Figure 2. 

The values for non-carcinogenic (HQ) risk associated with exposure to benzene in the cities of Torino, Perugia, and Lecce are shown in Table 4. HQ was <1 in all monitoring stations and in both 2014 and 2015 since all the chronic exposure concentrations (EC) for benzene were lower than RfC (3.00 × 10^−2^ mg/m^3^).

The carcinogenic risk analysis conducted for benzene (Table 5) showed that ICR for children was greater than 1 × 10^−6^ in the city of Torino, with the highest value in Rebaudengo station, while in Perugia and Lecce, it was lower. For adults, the carcinogenic risk was greater than 1 × 10^−6^ in all the cities, with the highest values in Torino.

## 4. Discussion

In this study the data concerning the airborne concentration of PM_10_ and benzene in Torino, Perugia and Lecce, detected by the Regional Environmental Protection Agency’s monitoring stations located in each city, were acquired; the level of atmospheric pollution attributable to these contaminants was assessed comparing the data with the limits indicated in the Italian legislation; and the analysis of inhalation risk associated to benzene exposure was performed according to standard USEPA methodology. 

Data related to the concentration of airborne contaminants revealed that Torino is the most polluted among the cities involved in this study both for PM_10_ and benzene and confirm the high level of air pollution in this city documented in other studies [34,35,36,37]. 

In particular, with regards to PM_10_, the target value of the annual average stated in the legislation is equal to 40 µg/m^3^. This value was exceeded in Consolata and Rebaudengo stations (Torino) in 2015. In addition, for the same parameter, the legislation indicates a daily limit of 50 µg/m^3^, which must not be exceeded more than 35 times in a calendar year. In this case, all the stations located in Torino recorded a number of excesses over the limit both in 2014 and 2015. In Perugia and Lecce, the number of exceedances of the daily limit was lower than the maximum number allowed by the legislation in both years. The situation could be even more alarming if the WHO’s Air Quality Guidelines [29] are taken into account. Based on a systematic review of literature on adverse health effects of air pollution, WHO set at 20 µg/m^3^ the limit for annual average of PM_10_. In our study, all the monitoring station located in urban areas measured annual averages of PM_10_ above the WHO limit highlighting a potential risk for cardiovascular and respiratory diseases caused by chronic exposure.

In contrast, the concentration of benzene measured by all the monitoring stations in both years never exceeded the limit of 5 µg/m^3^ indicated in the legislation. In this case, WHO guidelines set no safe limit for benzene, as even very low concentrations are considered to be potentially toxic.

The correlation between benzene and PM_10_ could allow us to verify if vehicular traffic contributed to the concentration of these pollutants in urban environments. It is known that the exhaust fumes of motor vehicles contain high concentration of particulate and benzene [7,8] and that vehicular traffic is the main source of benzene in the urban environment [5,8]. Therefore, a strong correlation between PM_10_ and benzene indicates a high contribution of vehicular traffic to urban pollution. In our study, this correlation was more evident in the city of Torino than in the other cities and confirmed the high concern about the vehicular traffic in this city highlighted in other studies [38]. This finding seemed to be confirmed by the comparison of PM_10_ levels between the “traffic” and “background” stations. It highlighted in Torino a significant contribution of vehicular traffic in PM_10_ concentration with differences greater than 10 µg/m^3^ which, according to other studies [39,40], may lead to a significant increase of chronic diseases for long-term exposure.

With regard to the inhalation risk analysis derived from exposure to benzene of population living near the monitoring sites, we followed the USEPA recommendations based on “Inhalation Dosimetry Methodology” [30]. This methodology takes into account generic exposure parameters [33] for target population (i.e., resident children or adult) determined through a conservative approach. In addition, with regard to exposure concentration, it suggests using the UCL of the dataset including all measurements of pollutant concentration detected over a time period or in a limited area. Moreover, the USEPA recommends that, when estimating risk via inhalation, risk assessors should use the concentration of the chemical in air as the exposure metric (mg/m^3^) rather than inhalation intake of a contaminant in air based on inhalation rate and body weight (mg/kg-day). 

In our study, the non-carcinogenic risk for exposure to benzene in the years 2014 and 2015 was lower than the limit of acceptability (HQ = 1) as the chronic exposure concentration in all sites was lower than the safety threshold concentration (RfC) detected by toxicological studies and reported in the IRIS database.

Regarding the carcinogenic risk, it is important to stress that there is not an exposure threshold to carcinogens below which there is no risk of developing cancer during one’s lifetime. However, many studies and regulations consider 1 × 10^−6^ as the acceptability threshold for the ICR [41,42,43,44]. The analysis highlighted that in 2014 and 2015, the increased risk for children to have a cancer due to an exposure duration of six years was greater than acceptability threshold in the city of Torino, while in the other cities ICR was lower. For adults the increased risk to have a cancer due to an exposure duration of 24 years was greater than the acceptability threshold in all the cities with the highest values in Torino. Our results suggest that the benzene exposure from vehicular traffic at levels lower than the current European Union limit of 5 μg/m^3^ [28] may lead to an increased risk of childhood leukemia and adult lymphoid diseases. These results are consistent with some previously reported epidemiologic studies that examined the relation between exposure to benzene from motorized traffic and childhood leukemia risk [45,46,47] and with evidence concerning adult lymphoid leukemia and other lymphoid malignancies [10,48].

This work is not without its limitations. In particular, all the assumptions contained in the USEPA methodology are based on a conservative approach in order to protect human health from possible underestimated exposures. This inevitably leads to an overestimation of the actual risks. Factors that may lead to overestimation of risk include use of:(1) 95% UCL on the mean exposure concentrations, (2) default exposure assumptions, such as an exposure time of 24 h per day and exposure frequency of 350 days per year, and (3) conservative toxicity values. The benzene IUR, for example, is based on the high end of a range of maximum likelihood values and includes uncertainty factors to account for limitations in the epidemiological studies for the dose-response and exposure data [18].

There are also some factors that may have led to an underestimation of risk in our study results. This risk assessment was limited by the spatial and temporal series taken into consideration. For the estimated chronic exposure, we used two years of monitoring data to estimate exposures over a 24-year or six-year exposure period (respectively for adults or children) and 2–3 monitoring station per city with the risk to exclude periods or areas in which the air benzene concentration is higher than study data. 

## 5. Conclusions

The findings of this study suggest the need for emission reduction policies to preserve human health from exposure to air pollutants in urban areas. These measures should include local and urgent provisions, such as blocking traffic, and global preventive measures that can ensure long-term healthiness of the air, such as stricter land management policies (i.e., urbanization and industrialization control) or encouraging the production and use of low-emission vehicles (i.e., electric vehicles, hydrogen, etc.).

The toxicology approach used for health risk assessment could be a strategy useful for the management of air quality in urban areas affected by widespread pollution. In addition, results highlight the importance of revising the criteria used for setting the legal limits concerning air quality in urban areas because the current limits are not always sufficient to protect human health from continuous and long-lasting inhalation exposures to toxic substances.

## Figures and Tables

**Table 1:** List of monitoring stations and pollutants detected in the three study cities.

Cities	Station Name	Geographic Coordinates	Zone Type *	Station Type *	Monitored Pollutants
Torino	Consolata	N 45°04′33.300′′ E 07°40′40.896′′	Urban	Traffic	NO_x_, NO_2_, NO, CO_2_, SO_2_, CO, O_3_, PM_10_, Heavy metal ^1^, Benzene, PAH ^2^.
	Rubino	N 45°02′30.762′′ E 07°37′33.324′′	Urban	Traffic	NO_x_, NO_2_, NO, CO_2_, SO_2_, CO, O_3_, PM_10_, PM_2__.__5_, Heavy metal ^1^, Benzene, PAH ^2^.
	Rebaudengo	N 45°06′13.821′′ E 07°41′42.531′′	Urban	Traffic	NO_x_, NO_2_, CO_2_, SO_2_, CO, PM_10_, PM_2.5_ Heavy metal ^1^, Benzene, PAH ^2^.
	Druento-La Mandria	N 45°10′23.3′′ E 07°33′38.0′′	Rural	Background	NO_2_, NO, NO_x_, PM_10_, O_3_, Heavy metal ^1^, PAH ^2^
Perugia	Parco Cortonese	N 43°06′15.701′′ E 12°21′48.788′′	Urban	Traffic	NO_x_, NO_2_, NO, CO_2_, SO_2_, CO, PM_10_, PM_2.5_, O_3_, Benzene, PAH ^2^
	Fontivegge	N 43°06′19.065′′ E 12°22′32.131′′	Urban	Traffic	NO_x_, NO_2_, NO, CO_2_, CO, PM_10_, PM_2.5_, O_3_, Benzene, PAH ^2^
	Torgiano-Brufa	N 43°03′58.9′′ E 12°28′08.7′′	Rural	Background	NO, NO_2_, NO_x_, O_3_, PM_10_, PM_2.5_
Lecce	Garigliano	N 40°21′48.203′′ E 18°10′22.215′′	Urban	Traffic	CO, NO_2_, SO_2_, PM_2.5_, PM_10_, Benzene
	Libertini	N 40°21′07.110′′ E 18°10′33.952′′	Urban	Traffic	CO, NO_2_, PM_2.5_, PM_10_, Benzene
	S. M. Cerrate	N 40°27′35.0′′ E18°06′59.2′′	Rural	Background	CO, PM_10_, NO_2_, O_3_, SO_2_, PM_2.5_

^1^ Lead, Nickel, Cadmium, Arsenic; ^2^ Policiclic Aromatic Hydrocarbon; * as defined by respective Regional Agencies for the Environmental Protection.

**Table 2:** Annual average ± standard deviation (SD) and number of exceedances of the daily limit value of PM_10_ registered by monitoring station in the cities involved in the study.

Cities	Stations	Annual Average ± SD (µg/m^3^)		Excesess Daily Limit ^1^ (*n*)	
		2014	2015	2014	2015
Torino	Consolata	34.72 ± 22.18 *	40.03 ± 28.05 *	75	93
	Rubino	31.27 ± 22.38 *	36.49 ± 27.04 *	58	84
	Rebaudengo	38.53 ± 24.80 *	41.26 ± 25.30 *	94	99
	Druento-La Mandria	19.75 ± 14.80	23.58 ± 16.20	12	26
Perugia	Parco Cortonese	21.17 ± 12.33 *	27.75 ± 15.40 *	12	34
	Fontivegge	20.46 ± 12.43 *	22.33 ± 14.67	14	32
	Torgiano-Brufa	16.38 ± 10.24	23.77 ±15.11	4	19
Lecce	Garigliano	22.03 ± 10.85 *	23.22 ± 11.54	9	7
	Libertini	23.48 ± 13.19 *	25.37 ± 11.81	11	8
	S.M. Cerrate	20.48 ± 10.19	26.85 ± 12.27	6	7

^1^ Daily limit of 50 µg/m^3^ set in the Directive 2008/50/EC [28]; * level of PM_10_ significantly different (*p* < 0.01) than in background station.

**Table 3:** Annual average of benzene registered by monitoring station in the cities involved in the study.

Cities	Stations	Annual Avarage µg/m^3^	
		2014	2015
Torino	Consolata	2.027	2.130
	Rubino	2.182	2.067
	Rebaudengo	2.429	2.607
Perugia	Parco Cortonese	0.592	0.785
	Fontivegge	0.918	1.022
Lecce	Garigliano	0.954	0.968
	Libertini	0.851	1.090

**Table 4:** Non-carcinogenic risk (HQ) associated with exposure to benzene in Torino, Perugia, and Lecce.

Cities	Station	HQ (Benzene)	
		2014	2015
Torino	Consolata	0.07	0.08
	Rubino	0.08	0.08
	Rabaudengo	0.09	0.10
Perugia	Parco Cortonese	0.02	0.03
	Fontivegge	0.03	0.04
Lecce	Garigliano	0.04	0.04
	Libertini	0.03	0.04

**Table 5:** Increased cancer risk (ICR) associated with exposure to benzene in Torino, Perugia, and Lecce.

Cities	Station	ICR (Child)		ICR (Adult)	
		2014	2015	2014	2015
Torino	Consolata	1.50 × 10^−6^	1.60 × 10^−6^	5.99 × 10^−6^	6.39 × 10^−6^
	Rubino	1.58 × 10^−6^	1.53 × 10^−6^	6.31 × 10^−6^	6.14 × 10^−6^
	Rebaudengo	1.84 × 10^−6^	1.95 × 10^−6^	7.35 × 10^−6^	7.35 × 10^−6^
Perugia	Parco Cortonese	4.49 × 10^−7^	5.26 × 10^−7^	1.80 × 10^−6^	2.10 × 10^−6^
	Fontivegge	6.41 × 10^−7^	7.05 × 10^−7^	2.56 × 10^−6^	2.82 × 10^−6^
Lecce	Garigliano	7.34 × 10^−7^	7.34 × 10^−7^	2.93 × 10^−6^	2.99 × 10^−6^
	Libertini	6.67 × 10^−7^	7.80 × 10^−7^	2.67 × 10^−6^	3.12 × 10^−6^

## Abbreviations

The following abbreviations are used in this manuscript:

ARPA	Regional Agency for the Environmental Protection
AT	Averaging time
CA	Concentration in air
EC	Exposure Concentration
ED	Exposure duration
EF	Exposure frequency
ET	Exposure time
HQ	Hazardous Quotient
ICR	Increased Cancer Risk
IRIS	Integrated Risk Information System
IUR	Inhalation Unit Risk
MAPEC	Monitoring Air Pollution Effects on Children for Supporting Public Health Policy
PAH	Polycyclic Aromatic Hydrocarbon
PM	Particulate Matter
RfC	Reference concentration
UCL	Upper Confidence Limit
USEPA	United States Environmental Protection Agency
WHO	World Health Organization
